# COVID-19 Infection during Pregnancy: Risk of Vertical Transmission, Fetal, and Neonatal Outcomes

**DOI:** 10.3390/jpm11060483

**Published:** 2021-05-28

**Authors:** Marwa Saadaoui, Manoj Kumar, Souhaila Al Khodor

**Affiliations:** Research Department, Sidra Medicine, Doha P.O. Box 26999, Qatar; msaadaoui@sidra.org (M.S.); mkumar@sidra.org (M.K.)

**Keywords:** SARS-CoV-2, coronavirus, pregnancy outcomes, ACE-2 receptor, immune response, placental antibody transfer

## Abstract

The COVID-19 pandemic is a worldwide, critical public health challenge and is considered one of the most communicable diseases that the world had faced so far. Response and symptoms associated with COVID-19 vary between the different cases recorded, but it is amply described that symptoms become more aggressive in subjects with a weaker immune system. This includes older subjects, patients with chronic diseases, patients with immunosuppression treatment, and pregnant women. Pregnant women are receiving more attention not only because of their altered physiological and immunological function but also for the potential risk of viral vertical transmission to the fetus or infant. However, very limited data about the impact of maternal infection during pregnancy, such as the possibility of vertical transmission in utero, during birth, or via breastfeeding, is available. Moreover, the impact of infection on the newborn in the short and long term remains poorly understood. Therefore, it is vital to collect and analyze data from pregnant women infected with COVID-19 to understand the viral pathophysiology during pregnancy and its effects on the offspring. In this article, we review the current knowledge about pre-and post-natal COVID-19 infection, and we discuss whether vertical transmission takes place in pregnant women infected with the virus and what are the current recommendations that pregnant women should follow in order to be protected from the virus.

## 1. Introduction

Pregnancy is an important and “formative period” governed by series of interconnected molecular and cellular mechanisms aimed to promote maternal homeostasis and maintain an optimal fetal-placental interaction while supporting fetal growth [1,2]. Despite being tightly regulated, many factors/events can disrupt this balance and lead to adverse pregnancy outcomes [3,4,5], which may result in failing the pregnancy and in few cases of maternal death [3,4] As per the UNICEF (United Nation United Nations International Children’s Emergency Fund), one pregnant woman or newborn dies every 11 s worldwide [6]. This great risk on pregnant women and babies’ health increases dramatically during pandemics [7].

Pregnant women are considered one of the most susceptible groups in a population, as changes during pregnancy, such as decreased functional residual capacity as well as changes in cellular immunity, can increase the risk of serious illness in response to viral infections and the risk of vertical transmission [8]. Vertical transmission is defined as the possibility of transmission from a mother to her fetus during the antepartum and intrapartum periods or to the neonate during the postpartum period, and it can occur via the placenta, body fluid contact during childbirth, or through direct contact owing to breastfeeding after birth ^9^. During pregnancy, the placenta acts as a barrier set to avoid transmission of infectious pathogens from the mother to her fetus; however, some infectious agents can cross the placental barrier, leading, in some cases, to congenital infections [9,10].

SARS-CoV-2 is an evolving coronavirus that was declared a pandemic on 11 March 2020 by the World Health Organization [11]. Coronaviruses are enveloped, non-segmented, positive-sense RNA viruses belonging to the family *Coronaviridae* [12]. A qRT-PCR using a nasopharyngeal swab is proposed as the best form of COVID-19 screening and diagnosis, although findings can be impacted by the sampling process, viral load, and other technical challenges [13]. With about 89,048,345 confirmed cases and 1,930,265 deaths reported by the WHO at the time of writing this review, this virus is easily and quickly spreading in the community [11]. After the first nine months since the COVID-19 pandemic started, a total of 116 million births were registered, and around 57,786 pregnant women in the USA alone were infected with the virus, while 71 lost their lives [14,15]. Unfortunately, many countries have reported a second wave of COVID-19 [16,17,18] with higher frequency of pregnant and post-partum women being infected in the second wave compared to the first one [19]. With the increase in the availability of COVID-19 testing in the developed countries, it has become possible to test more subjects, which has led to an increase in the detection rates of positive cases [20]. More recently, the world has faced new COVID-19 variants that are more transmissible due to mutations on the spike protein ^21^. The B.1.1.7 variant and B.1.351 variant originated in the United Kingdom and South Africa, respectively [21]. Both strains are considered more contagious and can cause severe disease (up to 30 percent) compared to the first strain [21].

**Figure 1:**
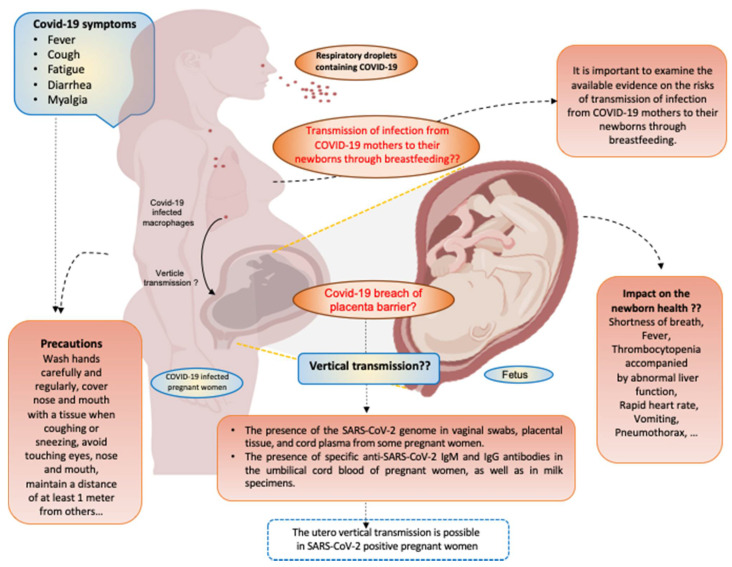
COVID-19 infection during pregnancy. Schematic model presenting the relationship between COVID-19 and pregnant women. Pregnant women acquire COVID-19 via respiratory droplets. COVID-19 is spread throughout the maternal vasculature and the symptoms appear after an incubation period of approximately 5.2 days. The most common symptoms of COVID-19 are fever, dry cough, fatigue, diarrhea, and myalgia. The impact of COVID-19 infection on pregnancy outcomes are not established. However, COVID-19 infection effects newborn health (shortness of breath, fever, and thrombocytopenia accompanied by abnormal liver function, rapid heart rate, vomiting, pneumothorax, etc.). So far, the vertical transmission from the woman to her baby may be possible, but no data related to the risks of transmission of COVID-19 infection through breastfeeding were recorded. Specific precautions (wash hands carefully and regularly, cover nose and mouth with a tissue when coughing or sneezing, avoid touching eyes, nose and mouth, maintain a distance of at least 1 m from others, etc.) are needed to protect both the mother and her baby. Figure was created with BioRender.com (accessed date 5 January 2021).

In this review, we assess the current knowledge about pre-and post-natal COVID-19 infection, and we discuss the prevalence, severity, and symptoms of COVID-19 infection and its related immune response during pregnancy. We also discuss the current literature describing whether vertical transmission takes place in pregnant women infected with the virus or not, as well as its potential effect on fetal and neonatal outcomes (summarized in Figure 1).

## 2. Symptoms of COVID-19 Infection during Pregnancy

In general, symptoms of COVID-19 occur after approximately 5.2 days of incubation [22]. Fever, dry cough, and fatigue are the most common symptoms of the infection, although other less common symptoms, including headache, nasal congestion, sore throat, body aches, conjunctivitis, skin rash, diarrhea, loss of taste or smell, and discoloration of fingers or toes, have been reported [23,24,25,26]. Fortunately, most of the reported positive cases of pregnant women showed only mild to moderate symptoms [27,28,29,30,31]. Based on data from the Mexican National Registry of Coronavirus [32], the comparison of COVID-19-related outcomes between 5183 pregnant and 5183 non-pregnant women with COVID-19 demonstrated that the percentage of death, pneumonia, and ICU admission were higher in pregnant women [32], suggesting that pregnancy significantly increases the risk of severe COVID-19 infections [33]. In addition, Black or Hispanic pregnant women are reportedly more affected by COVID-19 compared to others, and diabetic and obese pregnant women are at a higher risk of severe illness [32,34,35]. It is expected that infection by the SARS-CoV-2 virus during pregnancy may increase the risk of maternal and fetal health complications and evolve to severe pneumonia, causing admissions to intensive care units (ICU) [36].

## 3. Immune Response to COVID-19 Infection in Pregnancy

Special attention has been drawn to pregnant women and their infants in terms of the severity of COVID-19 infection and the possibility of its vertical transmission. Increase in the levels of inflammatory cytokines, such as IL-1, IL-2, IL-7, IL-10, granulocyte-colony stimulating factor, interferon-alfa-inducible protein 10, and tumor necrosis factor alfa, has been reported in blood, vaginal, and placenta samples collected from pregnant women [37,38]. High levels of these mediators can increase the severity of the inflammatory state in women, which may lead to pulmonary edema, severe hypoxia, respiratory failure, and multiple organ failure; in addition, it can cause a fall in T cells and an increase in leukocytes count as well as neutrophil-lymphocyte ratio [39,40]. All these factors have been associated with the severity of the disease and admissions to the ICU [41].

In contrast, others have suggested that the transition to a Th2 anti-inflammatory environment during pregnancy may result in protection from a severe COVID-19 presentation [42,43,44]. It has also been reported that COVID-19-positive pregnant women had reduced anti- COVID-19 IgG titers and were less likely to have detectable neutralizing antibodies compared to non-pregnant women [38].

Seroprevalence studies can detect infections in subjects that test negative on PCR (reviewed in [45]). Several studies were recently published to evaluate the progression of the seroprevalence of SARS-CoV-2 during pregnancy [46]. Cecilia et al. tested 769 serum samples obtained from routine serological testing during the first and third trimesters of pregnancy for specific IgG anti SARS-CoV-2 RBD and S proteins [47]. A high prevalence of COVID-19 was detected [46]. On the other hand, seroprevalence was similar between women in the first trimester of pregnancy and women in the third trimester, suggesting a similar risk of infection, but the proportion of women with symptoms and those who required hospitalization were higher in the third trimester group [47].

## 4. Vertical Transmission of SARS-CoV-2 and the Role of ACE-2 Receptor

**Figure 2:**
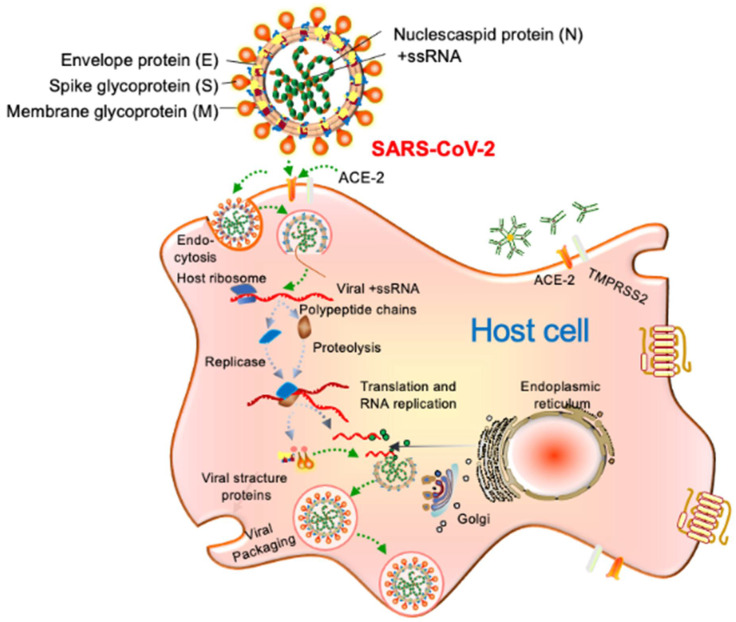
SARS-CoV-19 structure, host cell entry, and replication model. SARS-CoV-2 binds to host cells through the ACE-2 receptor, and, after uncoating, the components of virion use the host cell’s machinery to produce new viruses. Finally, the SARS-CoV-2 virions are released from the host cell by exocytosis.

To understand the vertical transmission and pathophysiology of SARS-CoV-2, it is important to discuss the viral structure, attachment to host cells, and replication cycle. A SARS-CoV-2 virion is a positive, single-stranded RNA virus with nucleocapsid and envelope, approximately 50–200 nm in diameter [45,46]. The virion has four structural proteins known as the S (spike), E (envelope), M (membrane), and N (nucleocapsid) proteins; the N protein holds the RNA genome, and the S, E, and M proteins together create the viral envelope ^12^. The spike glycoprotein-S facilitates the SARS-CoV-2 attachment to the angiotensin-converting enzyme-2 (ACE-2) receptor, and then serine proteases TMPRSS2 contribute to priming the S protein in order for it to fuse with the host cell membrane and replicate (Figure 2) [12,48].

Expression of the ACE-2 receptor on the surface of host cells is the hallmark of SARS-CoV-2 susceptibility and infection. To further understand the pathogenesis and vertical transmission of SARS-CoV-2, Jing et al. investigated the expression and activity of ACE-2 during pregnancy and found that the ACE-2 receptor is expressed on various cells, such as on the ovary, uterus, vagina, and on the placenta [49]. Moreover, Carole et al. assessed the ACE-2 expression in the placenta and compared the formalin-fixed placental tissues from 28 women who tested negative for COVID-19 infection as well as placental tissues from a COVID-19-positive woman during the second and third trimester of pregnancy [50]. They found a membranous expression of ACE-2 in the extra villous trophoblast as well as a strong and diffuse membranous staining of cytotrophoblast and syncytiotrophoblast cells of the placental villi [50]. They concluded that the expression of ACE-2 at the maternal–fetal interface is present throughout pregnancy regardless of the COVID-19 status [50].

While the expression of ACE-2 on the placenta supports vertical transmission, other routes of vertical transmission also exist. Recent reports provided evidence of its expression on the venous and arterial endothelium as well as in the smooth muscle of the umbilical cord, which further supports the possibility of COVID-19 vertical transmission [51]. In a recently published study [52], the investigators assessed the viral genome in nasopharyngeal swabs, vaginal swabs, maternal and umbilical cord plasma, placenta and umbilical cord biopsies, amniotic fluids, and milk from 31 mothers with SARS-CoV-2 infection [52]. Specific anti-SARS-CoV-2 antibodies and the expression of genes implicated in placental, maternal, and umbilical cord inflammatory responses were also studied [52].The presence of the SARS-CoV-2 genome was found in one umbilical cord blood and two at-term placentas, one vaginal sample, and in one milk sample [52]. In addition, unique anti-SARS-CoV-2 IgM and IgG antibodies were identified in one umbilical cord blood in pregnant women as well as in one breast milk specimen, and for the documented cases of vertical transmission, SARS-CoV-2 infection was accompanied by a strong inflammatory response [52].

Alzamora et al. reported a patient with a severe presentation of COVID-19 during pregnancy [53]. The patient underwent a cesarean section, and, immediately after delivery, neonatal isolation was performed without delayed cord clamping or skin-to-skin contact [53]. The neonatal nasopharyngeal swab collected 16 hours post-delivery was positive for COVID-19 [53]. A positive throat test obtained from a neonate born to a COVID-19-positive mother was recorded in another study by Wang et al. [51]. The placental and cord blood samples collected at birth were negative, but the baby had near contact with his mother early on, suggesting that the infection was through contact and not vertical transmission [51,54].

**Figure 3:**
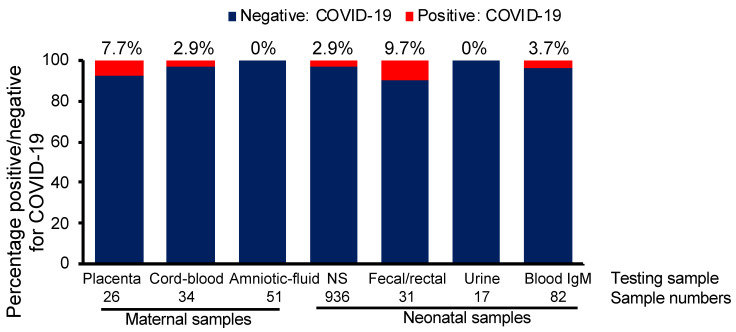
Vertical transmission rate of COVID-19 to neonates based on different testing sites and meta-data analysis of 38 studies. IgM, immunoglobulin M; NP, nasopharyngeal; COVID-19, coronavirus disease 2019.

Since the claim of vertical transmission in COVID19-infected mothers is not clear, we decided to analyze a total of 38 studies that assessed COVID-19 and pregnancy (also reviewed in [9]) by using various neonatal samples collected from mothers with COVID-19 infection. We concluded that vertical transmission of COVID-19 in the third trimester seems possible but occurs at very low frequency (Figure 3). The possibility of vertical transmission of COVID-19 was approximately 2.9% based on neonatal nasopharyngeal swab testing and 7.7% and 2.9% by placenta sample and cord blood analysis, respectively, while it was not evident based on amniotic fluid and neonatal urine analysis. In contrast, the highest vertical transmission possibility was observed at 9.7% based on neonatal fecal/rectal samples [9,55]. Although further studies are needed and no concrete assumptions can be taken because of the low number of cases analyzed, the possibility of vertical transmission remains likely, and testing babies born to COVID-19-infected mothers is vital [56].

## 5. COVID-19 Infection and Transplacental Antibody Transfer

The prevailing hypothesis is that antibodies against SARS-CoV-2 can cross the placenta during pregnancy, and several studies have focused on the transplacental antibody transfer [47,57]. In a recent study that enrolled pregnant women with SARS-CoV-2 infection, the authors assessed the SARS-CoV-2 antibodies against the receptor binding domain (RBD) on the S1 subunit of the spike protein and SARS-CoV-2 nucleocapsid (N) antigen using enzyme-linked immunosorbent assay [58]. The antibody analysis of 37 women with SARS-CoV-2 infection showed that only 65% of women showed an anti–receptor binding domain immunoglobin G, and 70% of them showed an anti-nucleocapsid antibody [58]. Additionally, the authors also quantified the SARS-CoV-2 antibodies in cord blood of 77 neonates and showed that only one newborn had detectable immunoglobulin M to the nucleocapsid [58]. This study suggested that the reduced transplacental transfer of anti- SARS-CoV-2 antibodies may predispose the neonates to COVID-19 infection.

Another study showed that while non-SARS-CoV-2-specific antibody transfer to the cord remains intact in COVID-19-infected mothers, SARS-CoV-2-specific antibody transfer is significantly lower in the third trimester of pregnancy, and this was related to a perturbation in the Fc glycosylation profiles [59]. Changes in the Fc glycosylation affect the capacity of the antibody to recruit innate immune effector functions aiming to control the pathogen [60]. On the other hand, Flannery et al. reported an efficient transfer of IgG antibodies from SARS-CoV-2-seropositive women and a positive correlation between maternal and cord antibody concentrations [57].

## 6. Role of COVID-19 Infection in Fetal and Neonatal Outcomes

The perinatal consequences of maternal infection with COVID-19 are variable, including premature labor, fetal distress, respiratory distress, thrombocytopenia accompanied by impaired liver function, and even death [61]. While maternal and neonatal mortality are rare [62], there is a considerable proportion of women that required ICU due to complication of COVID-19 infection [62]. Previous studies revealed that samples of cord blood, amniotic fluid, placenta, throat swabs collected from neonates, vaginal fluid, and breastmilk samples from subjects infected with COVID-19 and their babies have all been screened negative for the virus [63].

Zhu et al. reported the clinical characteristics and findings of 10 neonates (including 2 twins) born to 9 mothers with confirmed COVID-19 infection in 5 hospitals from January 20 to February 5, 2020 [61]. Those included shortness of breath (*n* = 6), fever (*n* = 2), thrombocytopenia accompanied by disrupted liver function (*n* = 2), rapid heart rate (*n* = 1), vomiting (*n* = 1), and pneumothorax (*n* = 1) [61]. Of these, five neonates were cured and released, one died because of multiple organ failure, and four neonates remained in a healthy state in the hospital [61]. Pharyngeal swab specimens were gathered to test for COVID-19 from 9 of the 10 neonates 1 to 9 days after birth, but all of the samples showed negative findings [61]. Nine infants (ranging from 1 month to 11 months) diagnosed with COVID-19 were tested in another study [64]. Of the nine children, four had fever, two had minor upper respiratory symptoms, one was asymptomatic, and there was no detail on symptoms available for the other two [64]. The time between admission and diagnosis was 1–3 days. All nine babies had at least one infected family member, and the baby’s infection usually happened after the infection of the family member [64]. All nine infants did not require intensive care or artificial ventilation and had no significant complications [65].

The main adverse neonatal outcome found is iatrogenic preterm birth, low birth weight, and admission to the NICU [62]. Chen et al. reported a 20% preterm birth incidence (out of 118 pregnancies), but no vertical transmission [66]. The current evidence suggest that neonates and children do not to develop severe COVID-19, and there is no evidence for congenital abnormalities associated with maternal infection’ however, uncovering neonatal infection is very important, as neonates can remain asymptomatic and serve as a reservoir to COVID-19 contributing, in turn, to community-related infections.

## 7. Risks of Transmission of SARS-CoV-2 via Breastfeeding

Transmission of viral infections through breastfeeding is well documented for cytomegalovirus, human immunodeficiency virus, and hepatitis B, among others [67]. The current data on the risks of viral transmission from COVID-19-infected mothers to their newborns via breastfeeding is relevant to review. However, the current evidence is very limited, with only a few smaller case series and case studies having been reported to date.

A systematic analysis conducted by Centeno–Tablante et al. included the scientific brief on breastfeeding and COVID-19 released by the WHO on 23 June 2020, revealing that out of 46 COVID-19-positive mothers whose breastmilk samples were checked for SARS-CoV-2, 43 breastmilk samples tested negative for SARS-CoV-2 [68]. Out of the three mothers whose breastmilk tested positive, only one infant tested positive for the virus, although it was not possible to ascertain the path of infection (breast milk or near contact) [68]. All seven colostrum samples obtained from seven mothers who tested positive for SARS-CoV-2 by PCR on a nasopharyngeal swab were negative for COVID-19 in another report by Marin et al. [69]. Another Italian study analyzed the expressed breast milk samples collected from two mothers who tested positive for COVID-19, and the virus was not detected by RT-PCR [70]. A recent systematic analysis was performed to examine existing evidence relevant to the existence of SARS-CoV-2 in COVID-19-infected pregnant women’s breast milk [71]. Eight studies investigating the existence of SARS-CoV-2 RNA in breast milk in 24 COVID-19-positive pregnant women during the third trimester of pregnancy were reported [71]. Biological tests obtained from the upper respiratory tract (throat or nasopharyngeal) of the neonates and placental tissues collected shortly after birth demonstrated negative findings for the presence of SARS-CoV-2 by RT-PCR [71].

However, one study reported the virus in breastmilk samples obtained from one mother who tested positive for four consecutive days before testing negative [72]. In 64 breast milk samples from 18 infected mothers, Chambers et al. analyzed COVID-19; only one breast milk sample was positive for the virus, but the same sample tested negative 2 days earlier and 12 and 41 days later, respectively [73].

Breastmilk samples obtained from mothers infected with COVID-19 tested negative for the virus, but it remains unknown whether the disease was spread via breastfeeding, close contact, or transmission in the few reports where the virus was found in breastmilk and the babies were diagnosed with COVID-19 infection as well.

## 8. Conclusions

One of these controversial topics that has been unknown to date is COVID-19 vertical transmission. Primary findings suggested no possibility of transmission from mother to fetus, while few recent studies indicated that vertical transmission may occur. The latest literature based on either restricted case studies or small cohort size indicates that it might be possible to vertically transmit the virus from the mother to her baby. In brief, the effect of infection with COVID-19 on the outcomes of pregnancy or health of the offspring is not well known.

In order to confirm the possible intrauterine vertical transmission, further research on larger cohorts of pregnant women in the first or second trimester is needed. Longitudinal studies to assess the long-term effect on babies born to COVID-19-infected mothers are also needed. As newborn babies may catch the virus after delivery, it is also a consideration to temporarily isolate a COVID-19-positive mother from her child, while on the other hand, recommendations from scientific societies advise to provide safety for the newborn baby without compromising the benefit of early contact with the mother. For these reasons, specific precaution measures need to be implemented to save the health and life of the mother and the baby. She should follow the same recommendations as nonpregnant women to minimize the exposure to the virus, including washing hands regularly; covering her nose and mouth with a tissue when coughing or sneezing; avoiding touching her eyes, nose, and mouth; and maintaining social distance.

As the possibility of vertical transmission is very significant in terms of safety and disease prevention for neonatologists and neonatal health care providers, vertical transmission of COVID-19 is a tremendous concern for both patients and caregivers. It is also strongly recommended to inform expectant mothers about the disease process as well as the measures needed to avoid and/or minimize the spread of COVID-19 to their newborns through skin contact. It is also necessary to use the best therapy for pregnant women with COVID-19 infection, taking into consideration those that will not present any harm to the babies’ growth and development. Studies in humans will take a long time to conclude, but preclinical studies using animal models will accelerate our knowledge.

## Data Availability

Not applicable.
